# Inflammatory cytokines during a manic episode in bd patients and its correlation with cognitive and affective symptoms at follow-up

**DOI:** 10.1192/j.eurpsy.2021.1660

**Published:** 2021-08-13

**Authors:** M. Estudillo Guerra, G. Torres Suarez, I. Mesia-Toledo, K. Pacheco-Barrios, J. Ramírez-Bemúdez

**Affiliations:** 1 Research Institute, Spaulding Rehabilitation Hospital and Massachusetts General Hospital, Harvard Medical School, Charlestown, United States of America; 2 Psychiatry, Centro Integral de Salud Mental, Mexico City, Mexico; 3 Neuromodulation Center And Center For Clinical Research Learning, Spaulding Rehabilitation Hospital and Massachusetts General Hospital, Harvard Medical School, Charlestown, United States of America; 4 Unidad De Investigación Para La Generación Y Síntesis De Evidencias En Salud, Vicerrectorado de Investigación, Universidad San Ignacio de Loyola, Lima, Peru; 5 Neuropsychiatry Unit, National Institute of Neurology and Neurosurgery, Mexico City, Mexico

**Keywords:** cytokines, manía, cognition, bipolar disorder

## Abstract

**Introduction:**

Bipolar Disorder (BD) is a severe recurrent disorder with a complex biogenetic and psychosocial etiology. The immune system cytokines in interaction with the CNS play a role in the pathophysiology.

**Objectives:**

To compare inflammatory cytokines between BD patients and controls during the manic episode; additionally, compare these cytokines with psychiatric symptoms and cognitive performance during follow-up.

**Methods:**

We recruited 25 BD patients in mania with paired controls. We measured baseline IL-2, IL-4, IL-8, IL-10, GM-CSG, TNF- α, and TNF- γ in plasma. We used U-Mann-Whitney for group comparison and Spearman correlation between sub-group follow-up assessments and cytokines.

**Results:**

We found a significant difference in IL-6 between subjects and controls (figure 1). During the follow-up, we found a correlation with psychiatric symptoms, cognition, and cytokines during manic episodes (Table 1). Table 1. Follow-up Correlation with cytokines during a manic episode.

**Table:** 

BD follow-up N=8	Cytokine	MADRS	BPRS	SCIP-S	WMT-SCIP-S	PST-SCIP-S
	IL-10		(-) Rho=-.957 (p=<0.001 R2=0.14).			
	IL-4	(+) Rho=.78 (p=0.02 R2=0.09)				
	INF- γ			(+) Rho=.73 (p=0.03 R2= 0.48)	(+) Rho=.751 (p=0.032 R2=0.53)	(+) Rho=.737 (p=.037 R2=0.40)
	INF- α				(+) Rho=.887 (p=.003 R2=0.53),	(+) Rho=.830 (p=0.011 R2=0.59)

(+): positive correlation; (-): negative correlation. WMT: working-memory test, PST: Processing-speed test.

**Figure:**
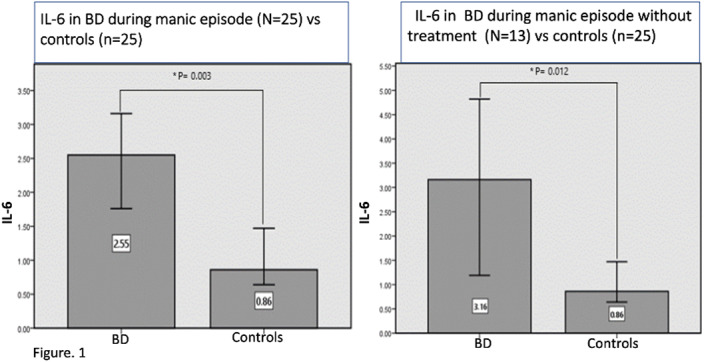

**Conclusions:**

IL-6 was significantly different in patients with BD during a manic episode regardless of the treatment they were taking. IL-10 at manic episode was negatively correlated to general psychiatric symptoms, IL-4 positive correlated to depressive symptoms, and cognitive performance was positively correlated to TNF- α and TNF- γ at follow-up.

**Disclosure:**

No significant relationships.

